# Targeting Focal Adhesion Kinase in Lung Diseases: Current Progress and Future Directions

**DOI:** 10.3390/biom15091233

**Published:** 2025-08-26

**Authors:** Ziyu Wan, Zefeng Zhu, Pengbin Wang, Xuan Xu, Tianhao Ma, Huari Li, Lexing Li, Feng Qian, Wei Gu

**Affiliations:** 1 Anhui Provincial Key Laboratory of Tumor Evolution and Intelligent Diagnosis and Treatment, Department of Biochemistry and Molecular Biology, Bengbu Medical University, Bengbu 233030, China; wzy@stu.bbmu.edu.cn (Z.W.); 20241002061@stu.bbmu.edu.cn (Z.Z.); wangcui268@gmail.com (P.W.); xuxuanxu077@gmail.com (X.X.); maxwellavyeg@gmail.com (T.M.); lihuari132@bbmc.edu.cn (H.L.); 2 College of Life Science and Technology, Wuhan University of Bioengineering, Wuhan 430415, China; llx1989@whsw.edu.cn; 3 Shanghai Frontiers Science Center of Drug Target Identification and Delivery, Engineering Research Center of Cell & Therapeutic Antibody, Ministry of Education, School of Pharmaceutical Sciences, National Key Laboratory of Innovative Immunotherapy, Shanghai Jiao Tong University, Shanghai 200240, China; fengqian@sjtu.edu.cn

**Keywords:** FAK, FAK inhibitors, lung cancer, ALI, pulmonary fibrosis, bronchial asthma

## Abstract

Focal adhesion kinase (FAK) is a crucial protein component of focal adhesions (FAs) and belongs to the cytoplasmic non-receptor protein tyrosine kinase family. FAK primarily regulates adhesion signaling and cell migration and is highly expressed in various tumors, including lung, liver, gastric, and colorectal cancers, as well as in conditions such as acute lung injury (ALI) and pulmonary fibrosis (PF). Recent research on FAK and its small-molecule inhibitors has revealed that targeting FAK provides a novel approach for treating various lung diseases. FAK inhibitors can obstruct signaling pathways, demonstrating anti-tumor, anti-inflammatory, and anti-fibrotic effects. In lung cancer, FAK inhibitors suppress tumor growth and metastasis; in ALI, they exert protective effects by alleviating inflammatory responses and oxidative stress; and in pulmonary fibrosis, FAK inhibitors reduce fibroblast activation and inhibit collagen deposition. The findings demonstrate promising efficacy and an acceptable safety profile in preclinical models. However, these early-stage results require further validation through clinical studies. Additionally, the underlying mechanisms, as well as the toxic effects and side effects, necessitate further in-depth investigation. Some have progressed to clinical trials (Defactinib (Phase II), PF-562271 (Phase I), CEP-37440 (Phase I), PND-1186 (Phase I), GSK-2256098 (Phase II), BI-853520 (Phase I)), offering potential therapeutic targets for lung diseases. Collectively, these findings establish a foundational basis for the advancement of FAK inhibitor discovery. Emerging methodologies, such as PROTAC degraders and combination regimens, demonstrate significant potential for future research. Based on a comprehensive analysis of the relevant literature from 2015 to the present, this review briefly introduces the structure and function of FAK and discusses recent research advancements regarding FAK and its inhibitors in the context of pulmonary diseases.

## 1. Introduction

Lung diseases, including lung cancer, ALI, chronic obstructive pulmonary disease (COPD), idiopathic pulmonary fibrosis (IPF), and asthma, pose a significant threat to global public health. Notably, lung cancer and PF are particularly concerning. Lung cancer ranks among the most prevalent and lethal cancers regarding both incidence and mortality [1], with over 1.8 million fatalities attributed to it annually [2].

PF is an interstitial lung disease (ILD) with an incidence rate ranging from 1 to 31.5 cases per 100,000 individuals [3,4]. Despite the high prevalence of this lung disease and its severe complications, the available treatment options remain limited. When using targeted therapy and immunotherapy to treat lung cancer, issues such as drug resistance and adverse effects may arise [5]. Pirfenidone and Nintedanib are drugs specific to IPF that are also effective for non-IPF fibrotic ILD. However, these two drugs exhibit different types and degrees of side effects, which increases the challenges associated with clinical treatment [6,7,8]. FAK, located on human chromosome 8, plays a crucial role as a phosphorylase kinase [9,10,11]. Extracellular signals are received through cell surface transmembrane receptors, which participate in intracellular signal transduction and play a pivotal role in cellular proliferation, migration, survival, and extracellular matrix (ECM) remodeling [12]. FAK has been identified as a potential therapeutic target for lung diseases due to its overactivation, which is closely associated with various pulmonary pathologies, including lung cancer (characterized by proliferation and migration), pulmonary fibrosis (marked by fibrosis), and acute lung injury (associated with inflammation) [13,14]. In these conditions, FAK is involved in regulating downstream signaling pathways that contribute to pulmonary inflammation, fibrosis, and the formation of the tumor microenvironment.

Currently, several FAK inhibitors, including PF-562271, Defactinib, and GSK2256098, have progressed to the clinical trial phase. Defactinib has demonstrated antitumor activity in non-small cell lung cancer (NSCLC) [15], whereas PF-562271 inhibits fibroblast activation and collagen deposition in PF, showcasing its therapeutic potential [16]. GSK2256098 effectively inhibits phosphorylation of the FAK kinase at the Y397 site in renal cancer, uterine cancer, and pancreatic ductal adenocarcinoma, thereby suppressing tumor cell proliferation, migration, and invasion [17,18,19]. FAK inhibitor PF-573228 reduces collagen I-induced hypo-contractility and alleviates asthma [20]. In a COPD mouse model induced by cigarette smoke extract (CSE), PTP1B overexpression significantly reduced p-FAK protein levels and alleviated lung tissue damage [21]. However, the clinical application of FAK inhibitors still faces numerous challenges, including drug selectivity, drug resistance, and potential toxic side effects. We reviewed the molecular mechanisms of FAK in pulmonary diseases and the latest research progress regarding its inhibitors. In the relevant literature from the past decade, FAK inhibitors have been extensively studied in lung cancer, less so in ALI, and there is an upward trend in their study related to PF. The investigation of FAK inhibitors in COPD remains an area that requires urgent exploration. This review focuses on the therapeutic potential of FAK inhibitors in lung cancer, ALI, PF, bronchial asthma, and COPD. This review is the first to summarize the role of FAK and FAK inhibitors in various lung diseases. Our aim is to uncover new treatment approaches for pulmonary diseases and to provide fresh insights into their management.

## 2. Method

### 2.1. Systemic Review

A systematic literature review was conducted using the PubMed (https://pubmed.ncbi.nlm.nih.gov/, accessed on 24 August 2025) and Web of Science (http://webofscience-clarivate-cn-s.vpndx.bbmu.edu.cn:8118/wos/woscc/basic-search, accessed on 24 August 2025) databases, with the keywords “FAK”, “FAK inhibitor”, “lung cancer”, “acute lung injury”, “pulmonary fibrosis”, and “chronic obstructive pulmonary disease”, focusing primarily on literature published from 2015 to the present. Additionally, we reviewed the references cited in the relevant literature. The final list of references was compiled based on the publication date of the articles and their relevance to our review.

### 2.2. Inclusion Criteria

- Research on FAK inhibitors in lung diseases.
- Peer-reviewed articles.
- Reviews, clinical trials.
- Provide complete data that can be accessed.

### 2.3. Exclusion Criteria

- Publications related to FAK inhibitors but not pulmonary diseases.
- Publications not in the English language.
- Lack of full-text availability.

## 3. FAK and Inhibitors

### 3.1. FAK Phosphorylation and Signaling Pathways

FAK is localized to FAs between cells and the ECM, playing a crucial role in cell survival and integrin-mediated cell migration. FAK can be recruited and phosphorylated during the early stages of cell adhesion, thereby mediating the formation of local adhesions [22]. FAK consists of three distinct domains, the N-terminal FERM domain, the kinase domain, and the C-terminal FAT domain [23]. These domains are separated by proline-rich regions, namely, PRP1, PRP2, and PRP3 [12], Specifically, PRP1 is situated at the N-terminal FERM domain, while PRP2 and PRP3 are located between the central kinase domain and the C-terminal FAT domain. FAK contains six tyrosine residues that are subject to phosphorylation: Y397, Y407, Y576, Y577, Y861, and Y925. Among these, Y397 serves as the primary phosphorylation site, which occurs between the FERM and kinase domains [24]. The interaction between the kinase domain of FAK and the FERM, FAT, and PR-I/PR-II regions can inhibit the phosphorylation of tyrosine 397 (Y397), thereby inducing self-inhibition of FAK [12]. Phosphorylation of Y397 is initiated by the binding of integrins to their ligands [24]. R-Ras plays a regulatory role in modulating the affinity and status of integrins [25]. The autophosphorylation of Y397 enhances FAK aggregation, as it serves as the binding site for the tyrosine kinase Src [26]. Following the phosphorylation of tyrosine 576 (Tyr576) and tyrosine 577 (Tyr577) mediated by Src, FAK is activated, resulting in a conformational change that prevents interaction between the N-terminal FERM domain and the FAK kinase domain [27].

**Figure 1:**
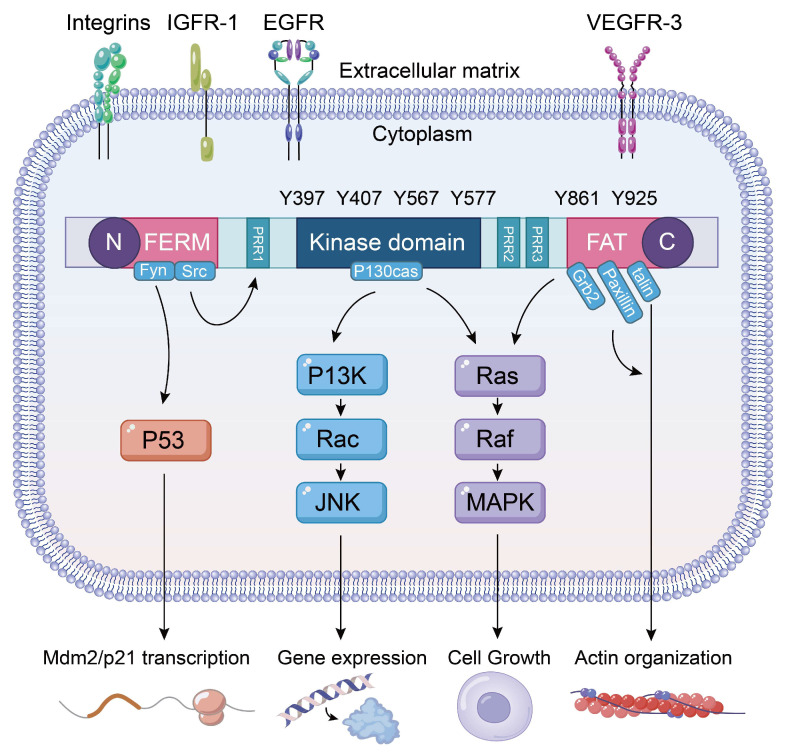
The schematic diagram illustrates the cellular signaling pathways involving FAK. It depicts the structure of FAK, highlighting the N-terminal FERM domain, which serves as the interaction site for integrins, growth factor receptors, and other molecules. The central kinase domain functions as the catalytic domain of the enzyme. The C-terminal region is identified as the protein interaction domain. Notably, Y397 serves as the primary site of autophosphorylation for FAK and is also crucial for mediating interactions with Src. The lower portion of the diagram illustrates the downstream signaling pathways regulated by FAK. FAK interacts with p53, facilitating FERM-mediated nuclear translocation of FAK and its subsequent binding to p53. This interaction promotes the binding of p53 to Mdm2, leading to the degradation of p53 through the ubiquitin-dependent pathway and the inhibition of apoptosis. Additionally, the FAK/Rac/JNK pathway is involved in the regulation of gene expression, while the FAK/MAPK pathway is critical for controlling cell proliferation and cell cycle regulation.

FAK receives various extracellular signals through transmembrane receptors located on the cell surface, including integrins, cytokines, growth factors, and G protein-coupled receptors [12]. It primarily activates four key signaling pathways: FAK-Src, FAK-PI3K, FAK-Ras-MAPK, and FAK-p53. The FAK-Src signaling pathway mediates downstream signaling of FAK by facilitating the binding and phosphorylation of p130^Cas^ through the FAK/Src complex [28,29,30,31,32]. FAK expression promotes the tyrosine phosphorylation of p130^Cas^ [33]. Disruption of FAK binding to Src or p130^Cas^ inhibits the multi-site phosphorylation of p130^Cas^ and consequently reduces cell migration [29,31,32]. The FAK/PI3K/AKT signaling pathway plays a crucial role in tumor development and progression. When stimulated by factors such as drugs, hypoxia, or oxidative stress, the PI3K/AKT signaling pathway is activated. FAK phosphorylation stimulates the activation of the downstream factor AKT, thereby promoting intracellular signal transduction [34,35,36]. The FAK/PI3K/AKT signaling pathway plays a crucial role in tumor development and progression. When stimulated by factors such as drugs, hypoxia, or oxidative stress, the PI3K/AKT signaling pathway is activated. FAK phosphorylation stimulates the activation of the downstream factor AKT, thereby promoting intracellular signal transduction. The FAK-Ras-MAPK signaling pathway is formed by a cascade reaction between FAK and mitogen-activated protein kinase (MAPK). Type II collagen, laminin-5, and other extracellular signals can activate the FAK-MAPK signaling pathway [37]. Activated FAK initiates Ras activation through two distinct pathways. Firstly, it phosphorylates tyrosine residues in proteins, leading to MAPK activation. Secondly, the adapter protein Grb2 binds to the phosphorylated Y397 residue within the Src-FAK complex [38]. Activated Ras binds to Raf, leading to the further activation of MAPKK, which in turn activates MAPK. MAPK translocates to the nucleus to regulate transcription, cell growth, proliferation, and differentiation [39]. The FAK-p53 signaling pathway interacts with p53 through the FERM domain, inhibiting both the activity of p53 and its transcriptional targets. Notably, p53 can bind to the FAK promoter, thereby inhibiting its luciferase activity [40]. p53 is predominantly localized in the cytoplasm [41], and the N-terminal region of FAK is found in the nucleus [42,43,44,45]. The interaction between p53 and FAK is crucial for signal transduction between the ECM and the nucleus (Figure 1).

### 3.2. FAK Inhibitor

Based on the FAK inhibition site and the type of inhibitors, these compounds can be classified into four categories, ATP-competitive inhibitors, FAK-FERM domain inhibitors, FAK-FAT domain inhibitors, and non-ATP-competitive inhibitors. ATP- competitive inhibitors demonstrate significant antiproliferative effects against various solid tumors [46]. FAK-FERM domain inhibitors impede the phosphorylation of Y397, while FAK-FAT domain inhibitors target the Y965 phosphorylation site [47]. Non-ATP-competitive inhibitors bind to allosteric sites, thereby disrupting specific protein interactions and enhancing the selectivity of FAK inhibition [48]. The ATP binding site within the kinase domain is specifically targeted by FAK inhibitors [12].

Most FAK inhibitors currently in clinical development are ATP-competitive inhibitors characterized by a common binding pattern at the FAK active site, including complexes such as FAK-TAE 226, FAK-PF473228, and PF-43196 [12]. Tyrosine kinase 2 (Pyk2), a non-receptor cytoplasmic tyrosine kinase, belongs to the FAK subfamily and shares 73% homology with FAK [49]. A critical aspect of developing FAK inhibitors is enhancing their targeting of Pyk2 [50]. In addition to selectivity issues, other obstacles include pharmacokinetic limitations and acquired resistance further, which further complicate the development of FAK inhibitors. Poor oral bioavailability and short half-life limiting target engagement [51]. FAK is overexpressed in lung cancer and promotes progression, metastasis, and drug resistance [52]. The activation of FAK signaling in EGFR-mutated NSCLC cells induces acquired resistance to EGFR tyrosine kinase inhibitors (EGFR-TKIs) [53]. Defactinib (VS-6063, PF-04554878) (IC_50_ = 1.5 nM), a second-generation FAK inhibitor developed by Pfizer Inc. in the United States, effectively inhibits both the kinase catalytic domain of recombinant FAK and endogenous FAK, as well as Pyk2 (IC_50_ = 2.4 nM) [54]. However, its selectivity for FAK kinase significantly exceeds that for Pyk2 [55]. Due to the homology between FAK and PYK2 in the kinase domain, high concentrations of Defactinib may partially inhibit PYK2 activity. PYK2 plays a role in the hematopoietic system. In hematopoietic cells, Pyk2 maintains the homeostasis of monocytes, regulates macrophage morphology and migration, controls B-cell and T-cell adhesion and function, and contributes to complement-mediated phagocytosis [56,57,58]. Off-target inhibition of PYK2 may lead to side effects associated with these tissues. In addition to inhibiting FAK activity, Defactinib modulates the tumor microenvironment and immune response, thereby suppressing tumor growth and metastasis. Currently, Defactinib is undergoing Phase II clinical trials and shows promising efficacy in the treatment of pancreatic cancer, prostate cancer, and NSCLC. The main toxic reactions associated with Defactinib include mild nausea, fatigue, headache, and a reversible elevation of unconjugated bilirubin resembling Gilbert’s syndrome [15,59,60].

PF-562271 is the first FAK inhibitor approved for clinical trials, demonstrating good drug ability and high selectivity [61]. It is a potent ATP-competitive inhibitor of FAK and Pyk2, exhibiting a selectivity for FAK (IC_50_ = 1.5 nm) that is nine times greater than that for Pyk2 (IC_50_ = 13 nM) [49]. Currently undergoing Phase I and Phase II clinical trials [62], PF-562271 has shown good tolerability and safety [63]. In a clinical trial involving 99 patients with advanced solid tumors, the maximum tolerated dose (MTD) and recommended Phase II dose (RP2D) of PF-00562271 were established at 125 mg twice daily with meals. The 150 mg dose was discontinued due to the occurrence of grade 3 headaches, nausea/vomiting, and edema [64]. It effectively blocks phosphorylation at the FAK Y397 site; inhibits the growth, migration, and invasion of ovarian and breast cancer cells; and induces apoptosis in tumor cell [65,66,67]. Additionally, PF-562271 alleviates polyinosinic-polycytidylic acid (Poly(I:C))-induced ALI and transfusion-related acute lung injury (TRALI) associated with the transfusion of aged platelets [68,69].

CEP-37440 (IC_50_ = 2 nM) is a novel orally active inhibitor developed by Teva and is currently undergoing Phase 1 clinical trials for patients with advanced solid tumors [70]. Anaplastic lymphoma kinase (ALK) is a transmembrane receptor tyrosine kinase, and its gene rearrangement is associated with NSCLC [71]. CEP-37440 is an effective dual inhibitor of FAK and ALK, exhibiting strong selective inhibitory activity against FAK (IC_50_ = 2 nM) and ALK (IC_50_ = 3.1 nM). CEP-37440 demonstrated favorable in vitro ADME properties and acceptable oral bioavailability in CD-1 mice, Sprague-Dawley rats, and cynomolgus monkeys [72]. The inhibition of FAK and ALK can effectively address drug resistance issues through a synergistic mechanism, thereby enhancing the therapeutic effect on tumors [73]. Furthermore, CEP-37440 possesses the capability to cross the blood–brain barrier, effectively inhibiting the formation of brain metastases [72]. VS-4718 (PND-1186) (IC_50_ = 1.5 nM) is an orally bioavailable pyridine compound that is highly selective for the ATP kinase domain. VS-4718 promotes caspase-3 activation by blocking FAK and p130^Cas^ tyrosine phosphorylation, which leads to apoptosis in breast cancer cells (4T1) and reduces tumor growth and metastasis in breast cancer [74].

GSK2256098 (IC_50_ = 0.4 nM) is an orally active ATP-competitive FAK inhibitor developed by GlaxoSmithKline, a UK-based pharmaceutical company. It has successfully completed Phase I clinical trials assessing safety, pharmacokinetics, and pharmacodynamics and has advanced to Phase II clinical trials. In a Phase I trial involving patients with advanced solid tumors, GSK2256098 demonstrated a manageable safety profile, with the majority of adverse reactions classified as Grade 1–2. The maximum tolerated dose (MTD) was established at 1000 mg administered twice daily [75]. GSK2256098 effectively inhibits the phosphorylation of the FAK kinase at the Y397 site in renal cell carcinoma, uterine cancer, and pancreatic ductal adenocarcinoma, thereby imepnding tumor cell proliferation, migration, and invasion [17,18,19]. Ifebemtinib (BI 853520 or IN10018) (IC_50_ = 1 nM) also targets the ATP active site of FAK. At a concentration of 0.1 μM, BI-853520 reduces autophosphorylation at the Y397 site, decreases FAK expression, and inhibits the proliferation of breast cancer cells [76]. Table 1 lists the FAK competitive inhibitors currently undergoing clinical trials.

Non-ATP-competitive FAK inhibitors achieve greater selectivity in FAK inhibition by binding to allosteric sites and disrupting specific protein interactions, such as the p53-FAK interaction. These are referred to as FAK allosteric inhibitors. The Vikas team has identified three potential allosteric inhibitors of the FAK protein as candidate drugs, MolProt-044-178-708, MolProt-005-909-736, and MolProt-044-811-051 [82]. The allosteric inhibitors BIRB 796, BAY 43-9006, and AAL-993 do not compete directly with ATP for binding. Rather, they exert their inhibitory effects by altering ATP binding dynamics [83]. The FAK FERM domain inhibitor interferes with the function of this domain inhibiting FAK activity by directly targeting the FAK activation site and preventing phosphorylation at the FAK Y397 site [84,85]. However, the FERM domain’s interactions with other proteins, such as membrane protrusions and integrins, along with the FAT domain’s extensive interaction interface with local adhesion spot proteins, complicate the design of high-affinity inhibitors.

## 4. FAK and Inhibitors in Lung Cancer

**Figure 2:**
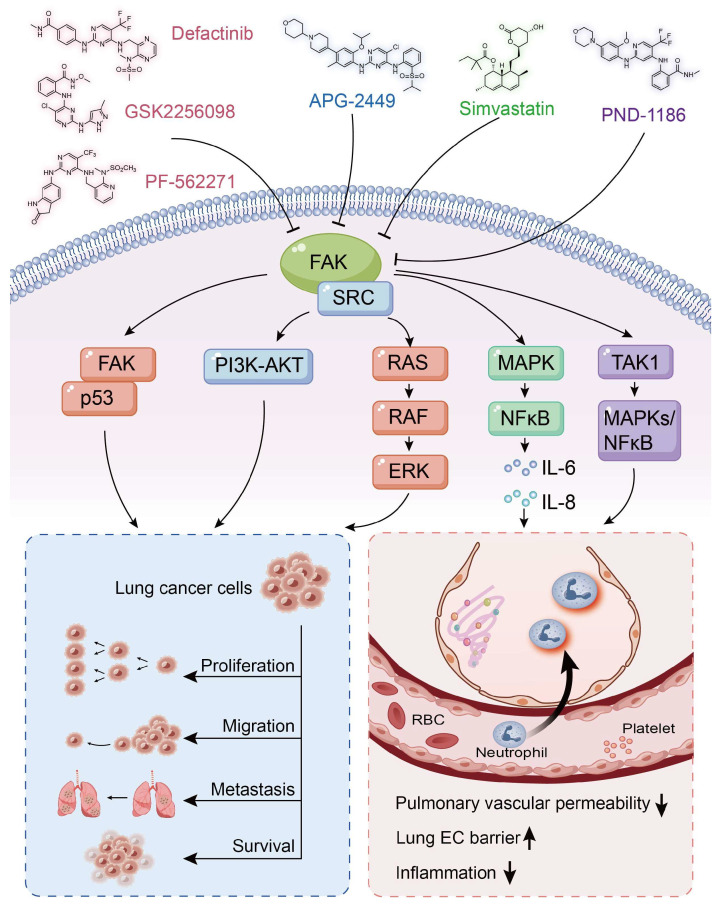
The mechanisms of action of five FAK inhibitors in lung cancer and ALI include Defactinib, GSK2256098, and PF-562271, which regulate tumor cell migration, proliferation, invasion, and survival through the FAK/p53 and FAK/RAS/RAF pathways. Additionally, APG-2449 modulates the migration, proliferation, invasion, and survival of NSCLC and ovarian cancer cell lines via the FAK/PI3K-AKT pathway. Furthermore, Simvastatin decreases the expression of inflammatory factors, such as IL-6 and IL-8, via the FAK/MAPK/NFκB signaling pathway. Additionally, it enhances lung barrier function and reduces pulmonary microvascular permeability. PND-1186 exerts anti-inflammatory effects through the FAK-TAK1-MAPKs/NFκB pathway, leading to reduced expression of inflammatory factors. Arrows indicate activation (→) and inhibition (⊣).

Lung cancer ranks as the second most prevalent cancer globally [86], categorized into two main types: small cell lung cancer (SCLC), accounting for 15%, and NSCLC, which constitutes 85% [87]. SCLC is characterized by its highly invasive nature, with cancer cells capable of metastasizing to the brain, liver, and bones [88]. In contrast, NSCLC typically progresses at a slower rate and is often diagnosed at an advanced metastatic stage [89]. The five-year survival rate following chemotherapy for lung cancer is approximately 25%. This relatively low survival rate can be largely attributed to the development of drug resistance in tumors, which diminishes the efficacy of chemotherapy [90]. Furthermore, FAK expression has been found to be significantly elevated in various lung cancer cell lines, including Calu3, LS277, and LS763 [91]. Notably, high levels of FAK expression correlate with disease staging and survival outcomes in NSCLC [92]. FAK is emerging as a promising therapeutic target, with its inhibitors playing a crucial role in lung cancer treatment. High levels of FAK expression correlate with various cancer types. Compounds such as Defactinib, GSK2256098, and PF-562271 modulate tumor cell migration, proliferation, invasion, and survival via the FAK/p53 and FAK/RAS/RAF signaling pathways (Figure 2). Defactinib, whether used alone or in conjunction with Avutometinib, effectively inhibits KRAS-mutated NSCLC through the FAK/AKT/MAPK pathway. In preclinical studies, the combination of Defatinib and Avutometinib effectively inhibits the growth of NCI-H358 tumors in mice, achieving a tumor reduction rate of up to 80% [93]. While epidermal growth factor receptor (EGFR) mutations are prevalent in NSCLC, the primary treatment method involves EGFR-tyrosine kinase inhibitors (TKIs). However, the development of TKI resistance poses significant challenges for long-term treatment. Studies have demonstrated that the combination of Defactinib and epidermal growth factor receptor tyrosine kinase inhibitor (EGFR-TKIs) can effectively overcome resistance. Defactinib inhibits the activation of downstream AKT/ERK pathways, thereby restoring sensitivity to EGFR-TKIs and enhancing their therapeutic efficacy in NSCLC cells. The combination of Defactinib and the EGFR-TKI gefitinib significantly reduces tumor volume in athymic nude mice bearing PC9GR tumors and decreases the expression levels of p-FAK, p-AKT, and p-ERK in tumor tissue [53]. Conteltinib (CT-707) specifically targets FAK and PYK2, exhibiting an IC50 value of approximately 1.6 nM for FAK [94]. Phase I clinical trials indicated that 83% of the 64 NSCLC patients treated with Conteltinib experienced tumor regression, with no significant occurrences of nausea, vomiting, or elevation in serum creatinine levels [95].

The FAK inhibitor APG-2449, with an IC50 value of 5.4 nM, demonstrated effective and sustained antitumor activity in NSCLC models when administered either alone or in combination with AZD-9291 and trametinib. APG-2449 regulates the migration, proliferation, invasion, and survival of both NSCLC and ovarian cancer cell lines through the FAK/PI3K-AKT signaling pathway [96]. PF-573228, an ATP-competitive inhibitor of FAK, inhibits phosphorylation at the Y576/Y577 site, thereby impairing its kinase activity [55,97]. Furthermore, PF-573228 effectively inhibits FAK activity in SCLC cell lines, reduces cell proliferation and DNA synthesis, induces G2-M phase arrest, and promotes cell apoptosis [98]. Ifebemtinib is a potent FAK inhibitor that demonstrates selectivity and efficacy against mesenchymal tumors [13]. The newly synthesized hybrid molecules (8a–h and 11a–h), which contain a 2,4-diphenylpyrimidine skeleton and coumarin groups, serve as novel FAK inhibitors with an IC50 value of 4.968 nM. These compounds can induce cell cycle arrest and apoptosis in H1299 cells, reduce the expression of matrix metalloproteinases MMP-2 and MMP-9, and inhibit the invasion and migration of NSCLC cells [99]. Diosmin, another FAK inhibitor, binds to FAK, disrupting the stability of Cyclin D1 and Snail, which leads to cell cycle arrest and inhibition of tumor metastasis. This mechanism demonstrates its anti-proliferative and anti-metastatic effects in lung adenocarcinoma [100].

## 5. FAK and Inhibitors in ALI/ARDS

ALI and acute respiratory distress syndrome (ARDS) represent severe clinical respiratory conditions [101,102]. The primary mechanism underlying the onset of ALI/ARDS is the disruption of the pulmonary microvascular barrier, which is driven by excessive inflammatory responses and increased permeability of both endothelial and epithelial cells. In the context of ALI, the response of endothelial cells (ECs) to mechanical and inflammatory stimuli is mediated by FAs, and alterations in endothelial cell barrier function are linked to specific phosphorylation events of FA proteins [103]. FAK regulates the disruption of EC barriers induced by reactive oxygen species (ROS). The inhibition of FAK not only reduces ROS production but also preserves barrier integrity, thereby underscoring the critical role of FAK in the pathogenesis of ALI [104,105]. Statins are widely recognized for their effective lipid-lowering properties and their capacity to decrease the incidence and mortality associated with coronary artery disease [106,107]. Simvastatin regulates inflammatory factors via the FAK/NFκB signaling pathway, protects the endothelial cell barrier, reduces pulmonary microvascular permeability, and alleviates inflammation, demonstrating a protective effect in an ALI animal model [105]. Furthermore, simvastatin can inhibit FAK expression through integrin β4, thereby preventing thrombin-induced endothelial cell barrier dysfunction in human pulmonary arteries. This suggests its potential role as a FAK inhibitor for the treatment of ALI [108].

The FAK inhibitor PF562271 mitigates ALI induced by Poly (I:C) by enhancing EC adhesion and barrier function [69]. Additionally, PF562271 demonstrates therapeutic efficacy in ALI resulting from aged platelet transfusion [68,109]. As a dual inhibitor of FAK and Pyk2, PF562271 improves survival rates in a mouse model of sepsis induced by cecal ligation and puncture (CLP); decreases the expression of pro-inflammatory factors such as TNF-α, IL-1β, IL-17, and IL-6; and alleviates inflammation as well as organ damage and dysfunction associated with sepsis [110]. PF-573228 inhibits the phosphorylation of FAK in rat pulmonary microvascular ECs, thereby mitigating thrombin-induced disruption of the endothelial barrier. In a lipopolysaccharide (LPS)-induced ALI model in mice, PF-573228 preserves EC barrier function by inhibiting FAK phosphorylation at Y397, highlighting its therapeutic potential for the treatment of mouse ALI [111]. Additionally, the FAK inhibitor PND-1186 reduces the expression of inflammatory factors, including TNF-α and IL-6, both in vivo and in vitro. In vivo, intravenous administration of 20 mg/kg PND-1186, administered 30 min prior to LPS injection, alleviates lung tissue damage, edema, and inflammatory infiltration. In vitro, PND-1186 inhibits LPS-induced FAK phosphorylation (Tyr397) and NF-κB activation in macrophages, blocks the FAK-TAK1 interaction, and inhibits the activation of the downstream MAPK/NF-κB pathway. Thus, PND-1186 presents a novel therapeutic approach for FAK inhibitor therapy in mouse models of ALI [112].

## 6. FAK and Inhibitors in Pulmonary Fibrosis

PF is a chronic lung disease characterized by the thickening of alveolar walls, impaired gas exchange, and restricted ventilation, which can ultimately lead to respiratory failure [113]. The progression of PF varies among patients and is primarily influenced by factors such as age, gender, and lung microbiota, as well as genetic and environmental influences [114,115,116]. ILD and IPF represent forms of irreversible and progressive pulmonary fibrosis [117,118]. The 5-year survival rate for patients with ILD ranges from 20% to 40% [119,120]. Pirfenidone and nintedanib are two commonly used pharmacological agents for the treatment of PF [6]. However, they are associated with notable adverse side effects, including gastrointestinal bleeding and severe diarrhea. Research indicates that FAK, produced by the protein tyrosine kinase 2 (PTK2) gene, plays a critical role in the pathogenesis of IPF. The processes of adhesion signaling and FAK activation are pivotal in the progression from lung lesions to fibrosis [121,122]. The development of fibrosis is linked to the ED-A splicing variants of fibronectin, endothelial-derived factors such as endothelin-1 (ET-1), matrix proteins like connective tissue growth factor (CCN2/CTGF), and alterations in matrix stiffness [123]. TGF-β serves as an inducer of lung myofibroblast differentiation, promoting the transformation of lung fibroblasts into myofibroblasts through FAK-mediated adhesion signaling [124]. In fibroblasts with FAK deficiencies, the ability for cell migration is significantly impaired; however, the re-expression of FAK can restore this migratory capacity [125].

Drugs targeting FAK are considered an effective strategy for combating fibrosis. In human fibroblasts, the FAK inhibitor PF-573228 effectively inhibits collagen production and induces apoptosis [126]. In mouse embryonic fibroblasts, FAK inhibition significantly reduces the expression of pro-fibrotic genes following stimulation with TGF-β or ET-1 [127]. Additionally, PF-562271 has been shown to prevent bleomycin-induced PF in mice without exhibiting toxic side effects. In mouse embryonic fibroblasts, PF-562271 inhibits ET-1 induced pro-fibrotic gene expression [16]. In models of bleomycin-induced PF, FAK knockout leads to a reduction in collagen network density, which corresponds to decreased collagen fiber arrangement and a less pronounced scar-like appearance. Furthermore, FAK knockout reduces the pro-fibrotic expression of *Acta2*, *Col1a1*, and *Tgfb1* genes, which are crucial for regulating the fibrotic response by modulating cell contractility, motility, and ECM deposition [128]. The FAK inhibitor TAE226 effectively inhibits the proliferation of MRC-5 cells in vitro and reduces the expression levels of α-smooth muscle actin (α-SMA) and type I collagen. Treatment with TAE226 significantly inhibited bleomycin-induced PF in mice, suggesting that FAK may serve as a potential therapeutic target for PF [129]. Given the complexity of the fibrosis and inflammatory response pathways in IPF, the irreversible lung damage it causes underscores the necessity for early intervention [128]. In bleomycin-induced IPF, the initial seven days are characterized by acute inflammation, marked by neutrophil infiltration and the upregulation of inflammatory factors. This is followed by a fibrotic phase after seven days, which involves the activation of fibroblasts and excessive deposition of extracellular matrix (ECM). Enhancing early inflammatory assessment and administering medication during this initial inflammatory phase can significantly improve treatment efficacy [130]. Moreover, the early administration of FAK inhibitors may lead to more favorable outcomes in mitigating fibrosis.

## 7. FAK and Inhibitors in Asthma and COPD

Asthma and COPD are chronic inflammatory diseases of the airways, affecting over 360 million and 544 million individuals, respectively [131]. Asthma is characterized by airway inflammation and hyperresponsiveness [132]. Both asthma and COPD exhibit similar pathological features, including the secretion of inflammatory cytokines by epithelial cells, increased mucus production, and the proliferation of airway smooth muscle (ASM) cells and pulmonary fibroblasts [133]. Elevated levels of YKL-40 (chitinase-like protein 1), a glycoprotein secreted by macrophages, neutrophils, and airway epithelial cells, have been observed in patients with asthma and COPD [134,135,136]. Furthermore, YKL-40 is correlated with asthma severity and serves as a potential biomarker for both conditions [137]. The phosphorylation of FAK is induced by YKL-40, which specifically activates the FAK/MAPK pathway in bronchial smooth muscle cells (BSMCs), resulting in muscle cell proliferation, hypertrophy, and subsequent thickening of the muscle layer. Inhibition of either the FAK or MAPK pathway effectively blocks the enhanced epithelial–mesenchymal transition (EMT) observed in Beas-2B (airway epithelial cells) in vitro and reduces fibrosis in an ovalbumin (OVA)-induced asthma model in vivo [138].

In asthma patients, the increased deposition of ECM protein collagen I around ASMs can induce a proliferative and low-contractile phenotype in these cells, which leads to enhanced ECM deposition and ASM hypertrophy, ultimately contributing to airway remodeling in asthma. The FAK inhibitor PF-573228 has been shown to inhibit collagen I-induced proliferation and reduce collagen I-induced low contractility [20]. Treatment of airway smooth muscle cells (ASMCs) with PF-573228 resulted in decreased mRNA levels and secretion of fibronectin, as well as the IL-13-induced eosinophil chemotactic factor-1 (eotaxin-1) and RANTES. This finding indicates that FAK plays a regulatory role in the production of ASMC cytokines induced by fibronectin, thereby contributing to asthma alleviation [139]. In human bronchial smooth muscle cells (BSMCs), isophorone diisocyanate (IPDI) has been demonstrated to induce BSMC proliferation and migration, which is associated with increased activation of FAK, Src, extracellular-signal-regulated kinase (ERK)1/2, and AKT. These findings suggest that FAK inhibitors may represent a promising class of drugs for asthma treatment [140]. Recent studies have shown that in a COPD mouse model induced by CSE, PTP1B overexpression significantly reduced p-FAK protein levels and alleviated lung tissue damage [21]. Interestingly, the tyrosine kinase activity of FAK is essential for preventing apoptosis [141]. FAK inactivation-induced anoikis represents a non-inflammatory mechanism of lung tissue destruction. In copper-deficient rats, reduced FAK phosphorylation in lung tissue is accompanied by increased levels of apoptosis markers such as caspase-3/8 and Bim, leading to alveolar structural damage (emphysema). Direct inhibition of FAK activity using FAK inhibitors (1,2,4,5-BT) or FAK siRNA similarly induces pulmonary emphysema and increases the expression of apoptotic markers. This mechanism may explain some cases of pulmonary emphysema in non-smokers (e.g., Menkes disease, environmental exposure) and provides new therapeutic targets for COPD treatment (e.g., activation of the FAK pathway) [142]. Smoking induces apoptosis of pulmonary endothelial cells and contributes to the development of pulmonary emphysema by downregulating FAK expression, in conjunction with factors such as VEGF and α-1-antitrypsin. The downregulation of FAK is directly linked to damage of the endothelial glycocalyx and destruction of alveolar structures, representing a common terminal pathway in the progression of pulmonary emphysema. Targeting FAK activation may present a novel therapeutic strategy for COPD, particularly in patients with smoking-related COPD [143].

## 8. Conclusions and Future Perspectives

Pulmonary diseases pose a significant threat to human health, and their treatment presents considerable challenges, necessitating the ongoing development and research of novel drugs that target specific pathways. FAK as a central effector molecule in the integrin signaling pathway, plays a critical role in the pathological progression of pulmonary diseases, and has emerged as a promising therapeutic target for their treatment. This review summarizes the current targeted FAK drugs and lists the FAK inhibitors that have entered clinical trials. In lung cancer, FAK inhibitors have been shown to inhibit tumor growth and metastasis. In ALI, FAK inhibitors exert a protective effect by alleviating inflammatory responses and oxidative stress. In PF, FAK inhibitors reduce fibroblast activation and inhibit collagen deposition. Notably, FAK inhibitors such as Defactinib, PF-562271, VS-4718, and GSK-2256098 have progressed to clinical trial phases (Defactinib Phase III, GSK-2256098 Phase II). Among these, PF-562271 demonstrates therapeutic effects in lung cancer, ALI, and PF. However, its role in asthma and COPD requires further investigation.

FAK inhibitors exert their effects through various signaling pathways in pulmonary diseases. In lung cancer, Defactinib, GSK2256098, and PF-562271 regulate tumor cell migration, proliferation, invasion, and survival via the FAK/p53 and FAK/RAS/RAF pathways, while APG-2449 operates through the FAK/PI3K-AKT pathway. In inflammatory lung diseases, statins modulate inflammatory factors through the FAK/NFκB signaling pathway, protect the endothelial cell barrier, reduce pulmonary microvascular permeability, and inhibit inflammation. PF-562271 has been shown to alleviate bleomycin induced PF in mice. In asthma, FAK can be activated by YKL-40, promoting airway smooth muscle proliferation, EMT, and fibrosis through the FAK/MAPK pathway. Inhibition of FAK reduces airway remodeling and mucus secretion. Further investigation into the role of FAK inhibitors in the progression of COPD could reveal new targets for drug development in pulmonary diseases. While FAK inhibitors alone exhibit limited therapeutic effects on pulmonary diseases, their combination with other treatments, such as Avutometinib, is considered to have significant research potential [93].

FAK inhibitors exhibit multifunctionality, with PF-562271 capable of simultaneously targeting tumor proliferation, metastasis, pulmonary fibrosis, inflammation, and barrier damage. In addressing treatment resistance in lung cancer, FAK inhibitors can enhance the efficacy of targeted therapies. For instance, Defactinib has been shown to reverse EGFR-TKI resistance in NSCLC. Additionally, some FAK inhibitors demonstrate good tolerability in clinical settings. In NSCLC patients, treatment with Conteltinib resulted in tumor regression in 83% of cases without severe side effects.

This review summarizes, for the first time, the research progress regarding various FAK inhibitors in the context of different lung diseases. FAK inhibitors have demonstrated potential in the treatment of lung diseases; however, they also present numerous limitations. Although several FAK inhibitors have progressed to clinical trial phases, the majority remain in preclinical or early clinical stages (Phase I/II). Many clinical trials involving FAK inhibitors are still in their infancy, characterized by small sample sizes and short follow-up durations, which complicates the assessment of their long-term efficacy and safety. Furthermore, in the case of lung cancer, tumor cells may compensate for the inhibition of FAK signaling by activating alternative signaling pathways. For instance, in KRAS-mutant non-small cell lung cancer, cancer cells that develop resistance to FAK inhibitors demonstrate enhanced ERK5-FAK signaling, which mitigates DNA damage and promotes tumor cell survival and proliferation [144]. FAK inhibitors encounter several challenges, including insufficient selectivity, unclear long-term safety, and complex mechanisms of resistance. Moving forward, it will be essential to develop precise targeting strategies, establish long-term models for toxicity evaluation, and implement combined interventions to address resistance.

PROTAC technology is an emerging cutting-edge chemical biology approach that utilizes heterodimeric small molecules to bring target proteins and E3 ubiquitin ligases into close proximity, thereby inducing polyubiquitination and subsequent degradation of the target proteins. The development of FAK degraders based on PROTAC technology signifies a novel direction in targeting FAK. By promoting FAK dysfunction through ubiquitination, the potential for drug resistance can be diminished [145,146,147]. Antibody–drug conjugates (ADCs) can specifically target tumor antigens, such as HER2 and TROP2, using monoclonal antibodies to deliver cytotoxic agents, such as MMAE and DM1, directly to tumor cells, thus minimizing systemic toxicity. The combination of FAK inhibitors and ADCs represents a pioneering research avenue in cancer treatment, where their synergistic effects may address the limitations of conventional therapies. Furthermore, it is imperative to advance clinical trials of FAK inhibitors in contexts such as acute exacerbations of COPD and radiation-induced lung injury, thereby providing new perspectives on the treatment of pulmonary inflammation and tumor diseases.

## Figures and Tables

**Table 1:** Clinical trial of FAK competitive inhibitors.

Chemical Compound/Company	Chemical Structure	Molecular Weight	Phase	IC50 on FAK	References
Defactinib (VS-6063)/Verastem	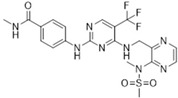	510.49	II	0.6 nM	[77,78,79]
PF-562271/Pfizer	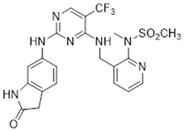	507.49	I	1.5 nM	[80]
CEP-37440/Cephalon	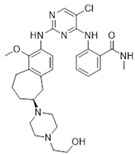	580.12	I	2 nM	[80]
VS-4718(PND-1186)/Verastem	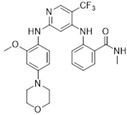	501.50	I	1.5 nM	[49]
GSK-2256098/GlaxoSmithKline	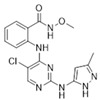	414.89	II	0.4 nM	[62]
BI-853520/Boehringer Ingelhein	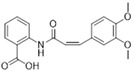	588.55	I	1 nM	[81]

## Data Availability

Not applicable.

## Abbreviations

The following abbreviations are used in this manuscript:

ADCs	Antibody–drug conjugates
ALI	Acute lung injury
ALK	Anaplastic lymphoma kinase
ARDS	Acute respiratory distress syndrome
ASM	Airway smooth muscle
ASMCs	Airway smooth muscle cells
BSMCs	Bronchial smooth muscle cells
CLP	Cecal ligation and puncture
COPD	Chronic obstructive pulmonary disease
CTGF	Connective tissue growth factor
CSE	Cigarette smoke extract
ECM	Extracellular matrix
ECs	Endothelial cells
EGFR	Epidermal growth factor receptor
EMT	Epithelial–mesenchymal transition
ERK	Extracellular-signal-regulated kinase
ET-1	Endothelin-1
FAK	Focal adhesion kinase
FAs	Focal adhesions
ILD	Interstitial lung disease
IPDI	Isophorone diisocyanate
IPF	Idiopathic pulmonary fibrosis
LPS	lipopolysaccharide
MAPK	Mitogen-activated protein kinase
NSCLC	Non-small cell lung cancer
OVA	Ovalbumin
PF	Pulmonary fibrosis
Poly (I:C)	Polyinosinic-polycytidylic acid
PTK2	Protein tyrosine kinase 2
Pyk2	Proline-rich tyrosine kinase 2
ROS	Reactive oxygen species
SCLC	Small cell lung cancer
TKIs	Tyrosine kinase inhibitors
EGFR-TKIs	Epidermal growth factor receptor tyrosine kinase inhibitor
TRALI	Transfusion-related acute lung injury
